# The Hospital World

**Published:** 1911-04-15

**Authors:** 


### THE HOSPITAL WORLD.

### PERSONALIA.

Great sorrow has been caused at the Leicester Infirmary by the sudden deaths of Miss Ivy Pick and Mrs. H. H. Woolley. The former, who was the victim of a taxicab accident in London, was the daughter of Mr. S. P. Pick, F.R.I.B.A., architect to the Leicester Infirmary, and by her talent for amateur theatricals had realised quite a sum for that institution, in respect of which she was recently elected to a life governorship. Mrs. Woolley was the wife of Mr. H. H. Woolley, the well-known organising secretary of the Leicester Saturday Hospital Society.

### Sir Charles Seely as Hospital President.

Sir Charles Seely has been elected president of the Nottingham General Hospital for the Coronation year. It is noteworthy that he held this post for the first time in 1873, and at his election the medical as well as the institutional staff referred to his great services to the hospital during this long connection. Sir Charles, who was born in 1833, was for many years the Parliamentary representative of Nottingham and High Sheriff of the County. He was a colonel of Volunteers for eighteen years, and is a Knight of Grace of St. John of Jerusalem.

---

Mr. David Painter McEuen, of Hayling Island and Pembridge Square, who left estate of the gross value of £404,093, has bequeathed £500 to St. Bartholomew's Hospital for the building fund, expressing the desire that his son Charles Henry be elected a governor; £500 each to the Royal Hospital for Incurables, Putney Hill, St. Mary's Hospital, Paddington, and the County and City of Perth Royal Infirmary; £200 to the Hillside Home Society for Incurables, Perth. He also left £2,000 to St. Bartholomew's Hospital, and £1,000 to St. Mary's Hospital. Should neither of his sons leave issue, he left, subject to their life interest, three-fifths of his residuary estate (which would amount to over £200,000) as to three-fourths thereof as follows: Five twenty-eighths to the Royal Hospital for Incurables, Putney, St. Mary's Hospital, Paddington, and the County and City of Perth Royal Infirmary; and two twenty-eighths to the Hillside Home Society for Incurables, Perth.

